# A magnet-actuated biomimetic device for isolating biological entities in microwells

**DOI:** 10.1038/s41598-018-31274-z

**Published:** 2018-08-24

**Authors:** Himani Sharma, Kimberley John, Anvesh Gaddam, Ambuja Navalkar, Samir K. Maji, Amit Agrawal

**Affiliations:** 1 Department of Mechanical Engineering, Indian Institute of Technology Bombay, Powai, Mumbai 400076 India; 2 Department of Biosciences and Bioengineering, Indian Institute of Technology Bombay, Powai, Mumbai 400076 India

## Abstract

Microwell platforms show great promise in single-cell studies and protein measurements because of their low volume sampling, rapid analysis and high throughput screening ability. However, the existing actuation mechanisms to manipulate the target samples and fabrication procedures involved in the microwell-based microfluidic devices are complex, resource-intensive and require an external power source. In this work, we present proof of concept of a simple, power-free and low-cost closed magnet digital microfluidics device for isolating biological entities in femtoliter-sized microwells. The target biological entities were encapsulated in magnetic liquid marbles and shuttled back and forth between micropatterned top and bottom plates in the microdevice to obtain high loading efficiency and short processing time. The microdevice performance was studied through fluorescent detection of three different entities: microbeads, bovine serum albumin (BSA) and *Escherichia coli*, captured in the microwell array. Almost 80% of the microwells were loaded with single microbeads in five shuttling cycles, in less than a minute. Further, a low volume of BSA was compartmentalized in the microwell array over a two order range of concentration. The microdevice exhibits two unique features: lotus leaf stamps were used to fabricate micropatterns (microwells and micropillars) on top and bottom plates to impart functionality and cost-effectiveness, and the target samples were actuated by a permanent magnet to make the microdevice power-free and simple in operation. The developed biomimetic microdevice is therefore capable of capturing a multitude of biological entities in low-resource settings.

## Introduction

Magnetic digital microfluidics is emerging as a potential choice for point-of-care applications due to its unique advantages over other digital microfluidic (DMF) techniques such as electrowetting-on-dielectric (EWOD), surface acoustic wave and light-driven^1^. Among the different actuation mechanisms in the magnetic digital microfluidics, magnetic liquid marbles show a great promise in manipulation of droplets due to their ease of formation and operation. Magnetic liquid marbles are created by encapsulating droplets with hydrophobic magnetic micro/nanoparticles and actuated through either a permanent magnet or an electromagnet. The magnetic particles assist in near-frictionless transportation on any surface avoiding the need for complex settings of DMF platforms. The solid particles form a shell around the droplet, reducing the area of exposed air-water interface, thus minimizing the evaporation of the droplet. Furthermore, magnetic forces enable reversible opening of shell of the magnetic liquid marbles. These remarkable features of liquid marbles were exploited in a few applications such as material transfer^2^, microreactors^3,4^, and electrochemical/optical detection^5,6^.

Hitherto, magnetic liquid marbles have mostly found application mainly in open DMF platforms i.e., operating droplets on a single-plate. However, it was shown that the closed (or two-plate) DMF platforms are capable of performing more versatile operations on droplets (such as moving, splitting and merging) compared to open DMF platforms, especially in EWOD-based DMF^7^. The closed EWOD-based DMF platforms were also successfully employed in isolation of bio-entities such as proteins and cells^8–10^. In these systems, bio-entities are generally encapsulated in droplets sandwiched between top and bottom plates containing microwells and electrodes, respectively. Isolation and capturing in microwells by labeling the target bio-entities for optical detection is one of the most promising approaches in measurement of low-abundant protein biomarkers and performing single-cell analysis^11^. For instance, microwell platforms enable access to the concentration regimes limited by the conventional ELISA (Enzyme-linked immunosorbent assay) technique. Furthermore, the closed DMF systems with microwells are capable of high throughput screening and enable large-scale integration and automation as compared to continuous flow systems^12^. Witters *et al*.^9^ demonstrated the functioning of a closed EWOD-based DMF platform to isolate streptavidin-coated superparamagnetic beads in microwells assisted by a permanent magnet, to detect single molecules of biotinylated β-galactosidase. A high loading efficiency (~98%) was achieved in isolating the single magnetic beads using a permanent magnet in a very short time as compared to gravity assisted loading of beads in microwells (~70%). Recently, Kumar *et al*.^10^ showed isolation of individual non-adherent yeast cells in microwells for single-cell analysis on a closed EWOD-based DMF platform.

In summary, the DMF allows consumption of relatively less reagent volume and is capable of performing more complex operations than the continuous flow microfluidic systems. Although, the magnetic DMF is less versatile than the EWOD-based DMF due to its inherent limitations, the former offers some unique advantages over the latter, particularly in reducing the complexity of the system, cost and ease of operation^13^. Further, the closed system approach of the EWOD-based DMF can be implemented with the magnetic DMF to mimic some of the applications mentioned earlier. One critical feature of microwell-based closed DMF systems is fabrication of femtolitre-sized small cavities on one of the plates. This volume corresponds to a cubic micrometer size of the cavity, therefore, fabrication of sub-10 µm features is an important step in designing these platforms. Photolithography has been extensively used to fabricate metallic or negative photoresist molds (eg. SU-8) with a feature size of less than 10 µm using laser or electron beam-written masks. The materials and equipment used in this step, however, eventually append to the cost of the device.

In contrast, our approach here is to avoid the use of such expensive manufacturing process for device fabrication. Nature comprises numerous examples of patterned surfaces, which evolved over very long period of time to adapt to relatively adverse conditions. For example, water-repellent plants such as *Colocasia esculenta* (taro), *Nelumbo nucifera* (lotus)^14^ and bactericidal wings of insect *Psaltoda claripennis* (Clanger cicada)^15^ have micro/nanometer-sized, multiscale textures over the surface, which could effectively serve as moulds for fabrication of microwells. Based on this approach, we design and demonstrate a handheld, low-cost and power-free microfluidic device for a wide variety of applications in biology. The simplicity and portability of the proposed microdevice will be particularly useful in low-resource environments for performing biological diagnostics. As a proof of concept, we demonstrate isolation of biological entities such as proteins, microbeads and bacteria in microwells using the developed microdevice.

## Proposed Design of the Microdevice

**Figure 1:**
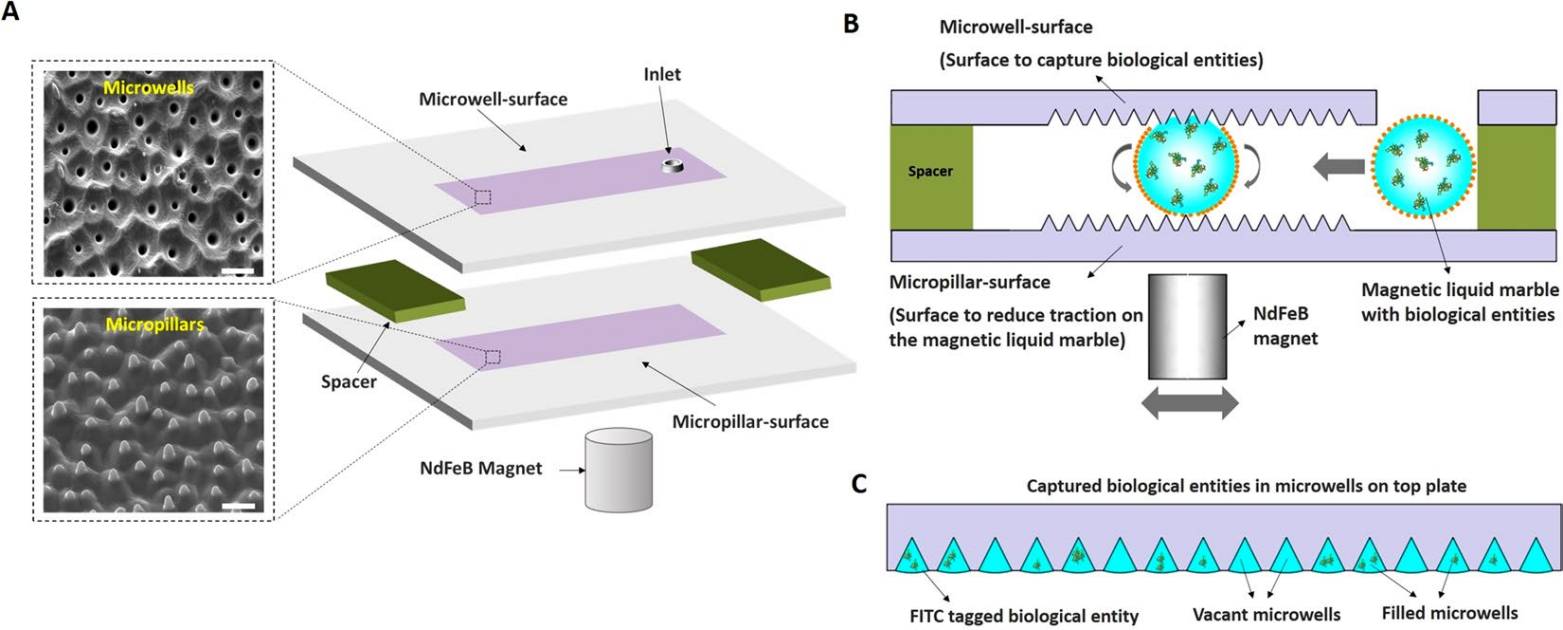
A magnetic actuated, biomimetic microdevice for isolating biological entities in microwells. **(A)** A schematic of the microdevice that integrates three layers: Top plate containing an array of microwells (inset, scale bar-25 µm), bottom plate containing an array of micropillars (inset, scale bar-25 µm), and spacers placed in between the top and bottom plates to form a chamber for movement of the magnetic liquid marble. **(B)** Functioning of the microdevice is shown. An opening of shell around the magnetic liquid marble along with the movement in the microdevice to allow capturing of biological entities in the microwells is shown. **(C)** A schematic representation of captured biological entities in microwells after the operation of the microdevice.

A simple low-cost microdevice for isolating biological entities in microwell is proposed in this work (Fig. 1A,B). The Fig. 1A, shows the proposed microdevice in which the top and bottom plates have plurality of microwells and micropillars, respectively. The spacer was placed in between the top and bottom plates for the formation of chamber. The top and bottom plate are arranged in such a way that the microwells and micropillars face each other inside the chamber of the microdevice. A cylindrical magnet was placed below the microdevice for actuating magnetic liquid marble. In this work, magnetic liquid marble refers to a Fe_3_O_4_ nanoparticle-cloaked droplet, which contains target biological entities to be captured. The functioning of the microdevice is illustrated in Fig. 1B. In the first step of operation, the magnet is moved towards chamber to enable partial opening of shell of the magnetic liquid marble. In the second step, the magnet will be moved back and forth along the chamber to allow the movement of magnetic liquid marble. The microwells on the top plate were used to capture the biological entities, which were encapsulated in the magnetic liquid marble (see Fig. 1C). On the other hand, the micropillars on the bottom plate reduce the frictional force, thereby responsible for smooth movement of the magnetic liquid marble.

In this microdevice, the surface topography of naturally available lotus leaf was exploited to prepare surfaces containing microwells and micropillars as shown in Fig. 1A (inset). The transfer of the features of lotus leaf using soft lithography circumvents expensive, time and resource-intensive conventional fabrication procedures, thereby rendering both cost-effectiveness and desired functionality to the microdevice. We further propose and demonstrate the use of or magnetic liquid marbles, such that the droplet movement can be controlled using a handheld permanent magnet. This obviates the need of sophisticated active fluidic delivery mechanisms involving complex attachments such as external pumps, controllers, power sources, and transducers. The utilization of the magnetic liquid marbles to manipulate biological samples further makes the proposed device to operate in a ‘power-free’ manner. As a result, the proposed microdevice is capable of capturing a multitude of biological entities in low-resource environments.

In section 3, characterization studies of as-synthesized magnetic nanoparticles employed in this work, and topography and wettability of the micropillar- and microwell- surfaces are presented. Subsequently, the role of surface wettability in improving the capturing efficiency of biological entities in microwells has been shown (section 4.1). Thereafter, it will be demonstrated that the micropillar-surface ensures that the rolling friction of magnetic liquid marbles is relatively low, thereby making the process energy efficient (section 4.2.2). The functioning of the microdevice and the mechanism of capturing is discussed in section 5. An application of the microdevice in isolating various biological entities is presented in section 6. The fabrication of various modules involved in the microdevice, are discussed in detail in the methods section.

## Characterization

In this section, characterization of the as-synthesized Fe_3_O_4_ nanoparticles for their size, shape and magnetic behaviour is presented. This is followed by an analysis of the morphology and wettability of plain and textured PDMS surfaces.

### Magnetic properties of Fe_3_O_4_ nanoparticles

**Figure 2:**
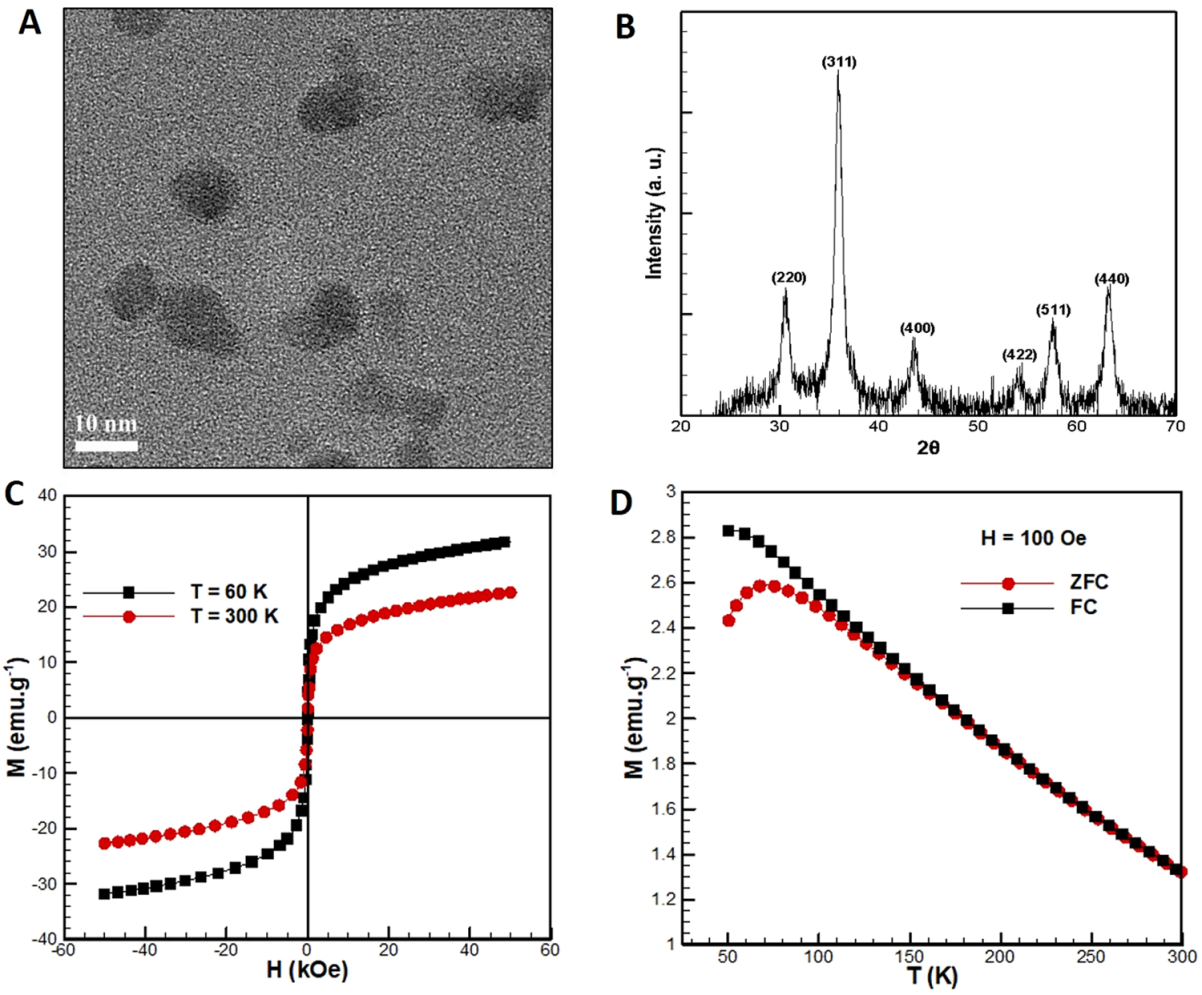
**(A)** TEM micrographs of the Fe_3_O_4_ nanoparticles. **(B)** XRD pattern of Fe_3_O_4_ nano particles. **(C)** Variation of magnetization of the Fe_3_O_4_ nanoparticles with the applied magnetic field at 300 K and 60 K. **(D)** Variation of magnetization of the Fe_3_O_4_ nanoparticles with temperature at 100 Oe with ZFC and FC.

Figure 2A shows the TEM micrographs of the Fe_3_O_4_ nanoparticles. The nanoparticles were found to be near-spherical with the characteristic size (or diameter) of about 9 nm. The XRD pattern of Fe_3_O_4_ nanoparticles is shown in Fig. 2B. The figure clearly shows the diffraction peaks of 2θ = 30.2, 35.6, 43.3, 53.7, 57.3 and 62.9, which can be indexed to planes (220), (311), (400), (422), (511) and (440) in a face-centered cubic Fe_3_O_4_, respectively. These are standard characteristics peaks, which are in well accordance with cubic spinel phase of Fe_3_O_4_. Further, the size (D) of crystallite/particle has been calculated using the Debye-Scherrer equation:

1 $${\rm{D}}={\rm{K}}\lambda /{\rm{\beta }}\,\cos \,{\rm{\theta }}$$

where K is the shape factor (0.9), λ is the X-ray wavelength (0.15418 nm), β is the full-width at half maximum (FWHM) value of diffraction lines, and θ is the Bragg angle. The estimated size of crystallites/particles was calculated to be 10.8 by using the diffraction peak (311).

The magnetization versus magnetic field (M-H) variation or hysteresis loop of the Fe_3_O4 nanoparticles is shown in Fig. 2C at 300 K and 60 K, respectively. It is apparent from the Fig. 2C that magnetic remanence is absent, indicating the superparamagnetic behaviour of the Fe_3_O_4_ nanoparticles. The saturation magnetization at an applied magnetic field of 50 kOe was 31.8 emu/g and 22.7 emu/g at 60 K and 300 K respectively. Figure 2D shows the magnetization versus temperature (M-T) variation in the range of 50–300 K in the applied magnetic field of 100 Oe with zero-field cooling (ZFC) and field cooling (FC), respectively. The kink in the M-T curve for ZFC represents the blocking temperature, which is observed at 75 K. As suggested by Park *et al*.^16^, the particle size obtained through M-T curve is around 9 nm, which is close to the value estimated by the TEM and XRD measurements.

### Topography and wettability of textured surfaces

**Figure 3:**
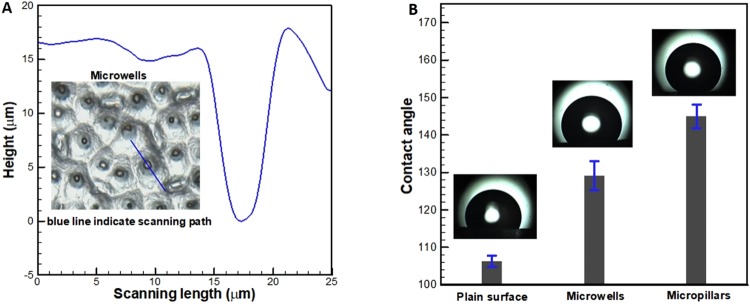
**(A)** Depth profile of a single microwell obtained by a 3D optical profilometer. **(B)** Variation of contact angle on plain and textured PDMS surfaces. (The error bars represent standard deviation of three experiment replicates, N = 3).

The 3D optical profilometer measurements revealed that the typical dimensions of the micropillars are 8–20 µm in height and 4–8 µm in base diameter. The Fig. 3A shows a typical profile and depth of one such microwell. The wettability of the textured PDMS surfaces was investigated with an interest to understand the degree of hydrophobicity by measuring the water contact angle. Figure 3B shows the variation in the contact angle on three surfaces viz., plain, microwell and micropillar PDMS surfaces. As the PDMS is intrinsically hydrophobic, any roughness on these surfaces increases the apparent contact angle as per the Cassie-Baxter’s theory^17^. The peculiar morphology of micropillars, i.e., combination of conical shape and hemispherical corner reduces the apparent liquid-solid contact area^18^. Therefore, surface with micropillars exhibited a higher degree of hydrophobicity than the surface with microwells.

## Device Optimization

In this section, we present optimization of individual modules of the microdevice such as top plate and bottom plate. The role of surface wettability on top plate containing microwells was investigated with an interest to enhance the isolation efficiency. The role of surface topography on the movement of the magnetic liquid marbles was also studied to reduce the rolling friction on the bottom plate.

### Mechanism of imbibition in microwells using surface modification

Formation of air bubbles is an inherent problem of hydrophobic surfaces containing textures such as micropillars, microwells, corners and traps, when an aqueous solution makes contact with the surface. These trapped air bubbles hinder the functionality of such textures through incomplete filling of microwells and reservoirs and avoid trapping of cells on the surface. One way to overcome this problem is by modifying the surface chemistry to induce hydrophilicity. This results in transition from the so-called Cassie-Baxter state to the Wenzel state and allows the solution containing biological entities to be fully in contact with the textured surface. While a few external simulating mechanisms to cause the Cassie-Wenzel transition (such as vibration, pressurization, and vacuum application) have been reported in the literature, a passive method to prevent air entrapment is clearly preferred as it removes the additional complexity involved with the aforementioned methods^19–21^.

Although PDMS is an attractive material for fabrication of microfluidic devices owing to its ease of handling, its hydrophobic nature causes non-specific adsorption of proteins. Therefore, it is necessary to render hydrophilicity to the PDMS surfaces containing microwells to enable complete filling, thereby enhancing isolation efficiency. A number of methods such as plasma oxidation^22^, thermal aging^23^, and surface chemical treatment^24^ were reported in the literature to impart hydrophilicity to PDMS surfaces. However, the surfaces modified by these methods either tend to recover hydrophobicity in a relatively short time or degrades the biocompatibility of the surface. In view of this, the microwell-surface was subjected to oxygen plasma treatment as explained in section B of Materials and Methods. The contact angle on as-prepared PDMS microwell-surfaces was measured to be 129.1° ± 3.8. After the oxygen plasma treatment, the contact angle on PDMS microwell-surfaces was reduced to ~60° ± 2.3. In this section, we will explain the mechanism of filling of liquid and liquids with solid sub-phases in microwells of different wettability. Subsequently, the experimental validation of theory will be demonstrated.

#### Theory

**Figure 4:**
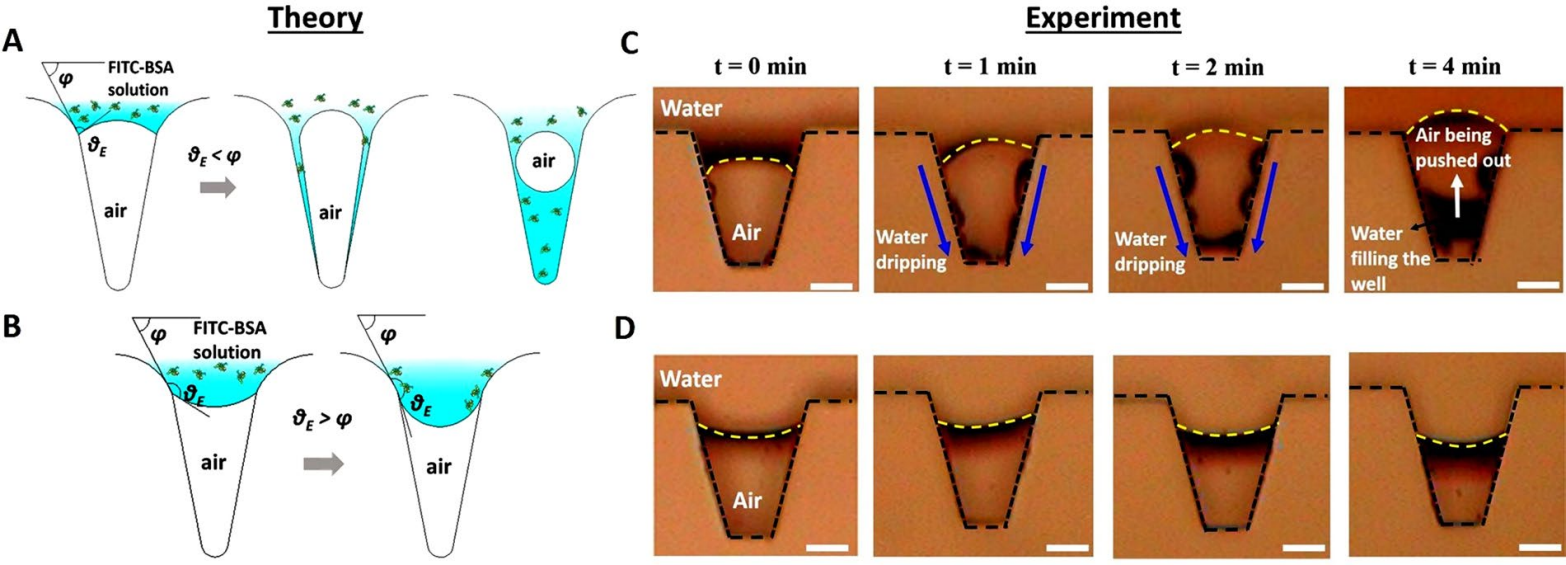
Schematic of mechanism of wetting in **(A)** hydrophilic microwells and **(B)** hydrophobic microwells, where *θ*_*E*_ is the equilibrium contact angle and *φ* is the angle made by the tangent at the highest point of cavity. Experimental results with DI water **(C)** showing the dynamics of water-air interface (yellow dashed line) and water dripping in hydrophilic microwells, and **(D)** pinning of contact line in hydrophobic microwells at different time intervals (scale bar −50 μm).

The mechanism of filling in hydrophobic and hydrophilic microwells, when a droplet of fixed volume containing solid-sub phases makes contact with the surface is illustrated in Fig. 4A,B. Under static conditions, with no external stimuli, the Cassie-Baxter state is only possible if *θ*_*E*_ > *φ*^25–27^, where *θ*_*E*_ is the equilibrium contact angle and *φ* is the angle made by the tangent at the highest point of the microwell. Note that, the shape of microwell considered here is based on the profile of a typical microwell (Fig. 3A).

Due to the dewetting nature of the hydrophobic surfaces, the liquid front of the droplet forms a convex interface into the microwell. The liquid- gas interface bows into the microwell and *θ*_*E*_ tends to increase over its previous state under any external stimuli, thus, the condition *θ*_*E*_ > *φ* will not be violated, as shown in Fig. 4B. This prolongs the existence of the Cassie-Baxter state for the surfaces with hydrophobic microwells, until the entrapped air completely dissolves into the solution. The solid sub-phase present in the aqueous solution migrate towards the inner surface of microwell with time, along the three-phase contact line.

Therefore, the solid sub-phase in the liquid eventually adheres to the periphery of the hydrophobic microwell due to electrostatic interactions. On the contrary, the liquid-air interface forms a concave shape for hydrophilic microwells due to their favourable wetting characteristics, thus violating the Cassie-Baxter condition *θ*_*E*_ > *φ*. The three-phase contact line advances along the inner walls of the hydrophilic microwells with time, leading to a complete wetting as shown in Fig. 4A. Similar observations were made through the Surface Evolver-based simulations in the recent work of Chang *et al*.^28^

#### Experimental validation

In order to assess the validity of the presented theory, two types of experiments were conducted. In the first one, experiments were conducted in microchannels with microwells (or trenches) arranged on the sidewalls to visualize the filling mechanism of liquid. To this end, we used a previously reported experimental approach^16^ (see supplementary material for more information). Briefly, we fabricated PDMS-based microchannels with microwells (similar to the shape of lotus leaf-based microwells, however, with the larger dimensions) on the sidewalls to allow visualization of dynamics of the liquid-air interface. Two sets of microchannels were considered with different wettability: hydrophobic and hydrophilic. To impart hydrophilicity, the PDMS films were treated with oxygen plasma before bonding. The process parameters were chosen in the same manner as mentioned in section 2.2. Deionized (DI) water was fed at the inlet of the microchannels through a syringe pump at a flow rate of 0.1 μL/min.

Once the microchannel was completely filled with DI water, the syringe pump was stopped and the outlet was closed with a plug. The pressure inside the microchannel was measured to be 11 mbar which is slightly higher than the atmospheric pressure. The air-water interface was visualized through an upright microscope fitted with a CCD camera. The snapshots of dynamics of the air-water interface for both the hydrophilic and hydrophobic microwells at different time intervals are shown in Fig. 4C,D. As explained earlier (see Fig. 4A), the favourable wetting characteristics of hydrophilic surface allows water to drip along the sidewalls of the microwells as evident from Fig. 4C. As the time progresses, the DI water which dripped along the sidewalls slowly replace the air and tend to settle on the bottom wall of the microwell. The air inside the microwells was pushed out into the outside bulk liquid over a period of time allowing the concave water-air interface to move towards the top as shown in the Fig. 4C. This leads to a complete filling of liquid in the hydrophilic microwells. On the other hand, the dewetting nature of the hydrophobic surfaces allows the convex water- air interface to be stabilized as shown in Fig. 4D, until an external stimuli is applied. This allows the solid sub-phases present in the liquid to settle on the periphery of the microwells.

**Figure 5:**
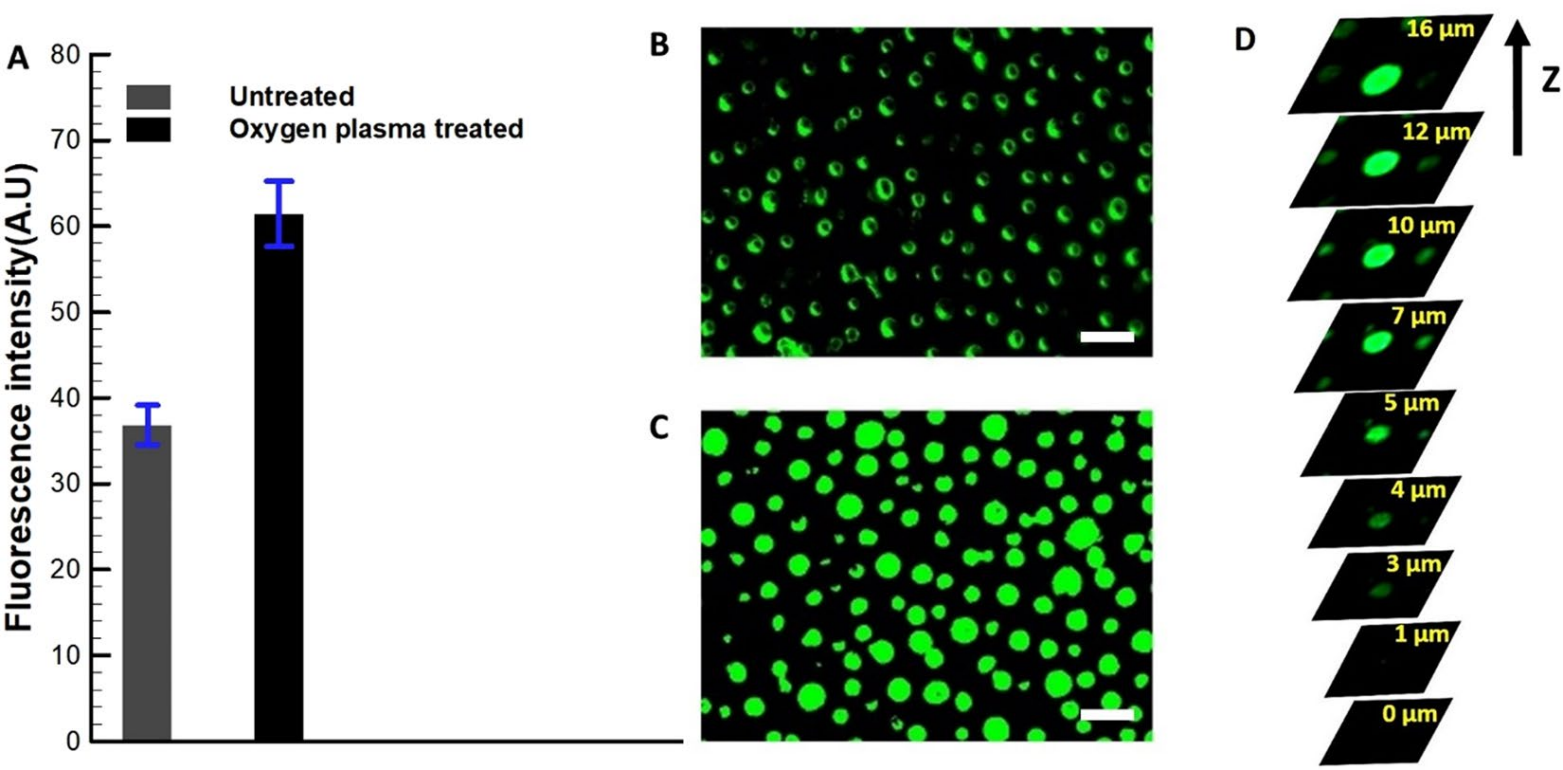
**(A)** Fluorescent intensity on both untreated and oxygen plasma treated PDMS microwell-surfaces. The corresponding fluorescent images for (**B)** untreated (hydrophobic, θ_CA_ = 129°) microwell-surface showing the aggregation of the FITC-BSA on the periphery of microwells and (**C)** oxygen plasma treated (hydrophilic, θ_CA_ = 60°) microwell-surface, showing the aggregation of the FITC-BSA in microwells (scale bar – 20 µm). **(D)** Micrographs obtained through confocal microscopy at different planes along the height of single microwell.

In the second set, experiments were conducted on the surfaces containing microwells to examine the filling mechanism of liquid with solid sub-phase. In this regard, the DI water droplets comprising the solid sub-phase (FITC-BSA) were placed on the hydrophilic and hydrophobic surfaces containing microwells. The droplet was allowed to rest on these surfaces for 5 minutes for the filling to takes place in the microwells and the remaining solution was collected back. Thereafter, the fluorescent intensity was recorded on the surfaces using fluorescent microscopy. It is evident from Fig. 5A, that more FITC-BSA was stacked inside the hydrophilic microwells than the hydrophobic microwells. The micrographs in Fig. 5B,C show that the FITC-BSA adsorption was restricted to the periphery of the hydrophobic microwells as described previously, whereas, it was completely filled in case of the hydrophilic microwells, as evident from the bright spots of fluorescent signal (The time-dependent study of imbibition in microwells is provided in supplementary material). Furthermore, the confocal microscopy was used to verify the filling of microwells. As it is evident from the sequence of images from the top surface of the microwell to its bottom surface in the Fig. 5D, the microwells are completely filled after surface modification. It is important to note that the Bond number (a non-dimensional number representing the ratio of the gravity to the surface tension force) is much less than unity, the orientation has no effect on the imbibition process of aqueous solution in the microwells. Therefore, these results will be equally applied for inverted microwell-surfaces in the microdevice as implied in Fig. 1B,C.

### Unit operations on magnetic liquid marbles

Here, we will discuss formation of magnetic liquid marbles, followed by their manipulation in terms of shell stripping and their movement on plain and textured surfaces with the aid of a permanent magnet.

#### Shell manipulation

**Figure 6:**
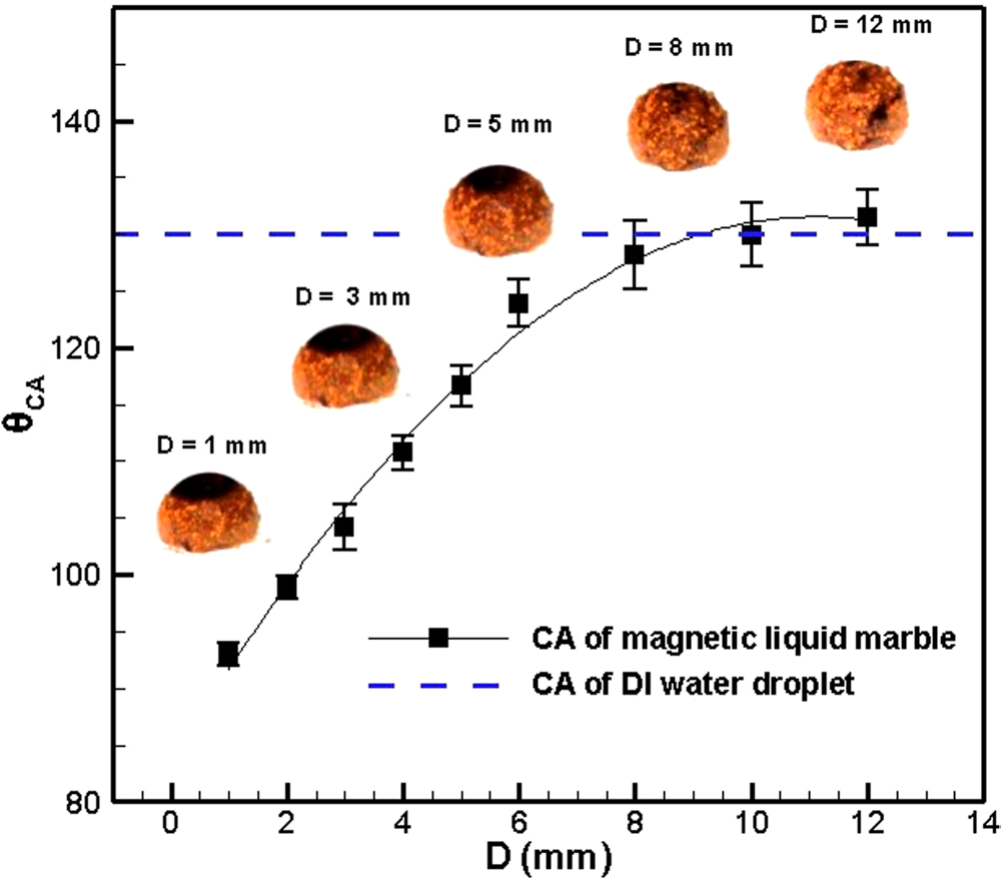
Variation of contact angle of the magnetic liquid marble with the distance from the magnet (the trend line and shell stripping images are included in the figure). Dashed line indicate the value of contact angle of DI water as control.

In order to create magnetic liquid marbles, initially DI water was pipetted in the form of a droplet onto a bed of Fe_3_O_4_ nanoparticles placed on a glass slide (surface coverage of droplet by nanoparticles is included in supplementary material). Then, the droplet was moved back and forth until the Fe_3_O_4_ nanoparticles self-assembled over the droplet surface. Once the droplet was completely cloaked with the Fe_3_O_4_ nanoparticles, the resulting magnetic liquid marble was placed on a Teflon-coated glass slide. The cylindrical magnet was fitted to a manual linear stage to vary the distance between the magnetic liquid marble and the magnet as shown in inset of Fig. 6. The variation in contact angle of the magnetic liquid marble was measured along with the visualization of shell stripping. Figure 6 shows the variation of the contact angle of the magnetic liquid marble with distance from the magnet (D). It is evident from Fig. 6 that the magnetic liquid marble deforms as the magnet moves towards the surface, resulting in a decrease in the contact angle. This is attributed the fact that surface tension forces along the periphery of the droplet were overcome by the force due to magnetic field on the Fe_3_O_4_ nanoparticles. At a larger distance i.e., when the intensity of magnetic field was less, the contact angle was measured to be 131.5° ± 2.5°. This indicates that the Fe_3_O_4_ nanoparticles were hydrophobic in nature. At the same time, the nanoparticles were found to cover the droplet surface, leaving very little area of the water-air interface exposed to the atmosphere for D ≤ 8 mm. The hydrophobic nature of the Fe_3_O_4_ nanoparticles allows them to stick only to the periphery of the droplet. As the magnet approaches towards the magnetic liquid marble, the shell made of Fe_3_O_4_ nanoparticles starts to fracture at different locations. A further movement of the magnet increases the magnetic field and allow continuous motion of the Fe_3_O_4_ nanoparticles along the periphery. This will help in opening the topmost portion of the magnetic liquid marble as shown in Fig. 6. Furthermore, the area of the exposed water-air interface was shown to increase with a decrease in the distance from the magnet. However, the shell stripping was only partially recovered with an increase in the distance of magnet from the magnetic liquid marble.

#### Motion manipulation

**Figure 7:**
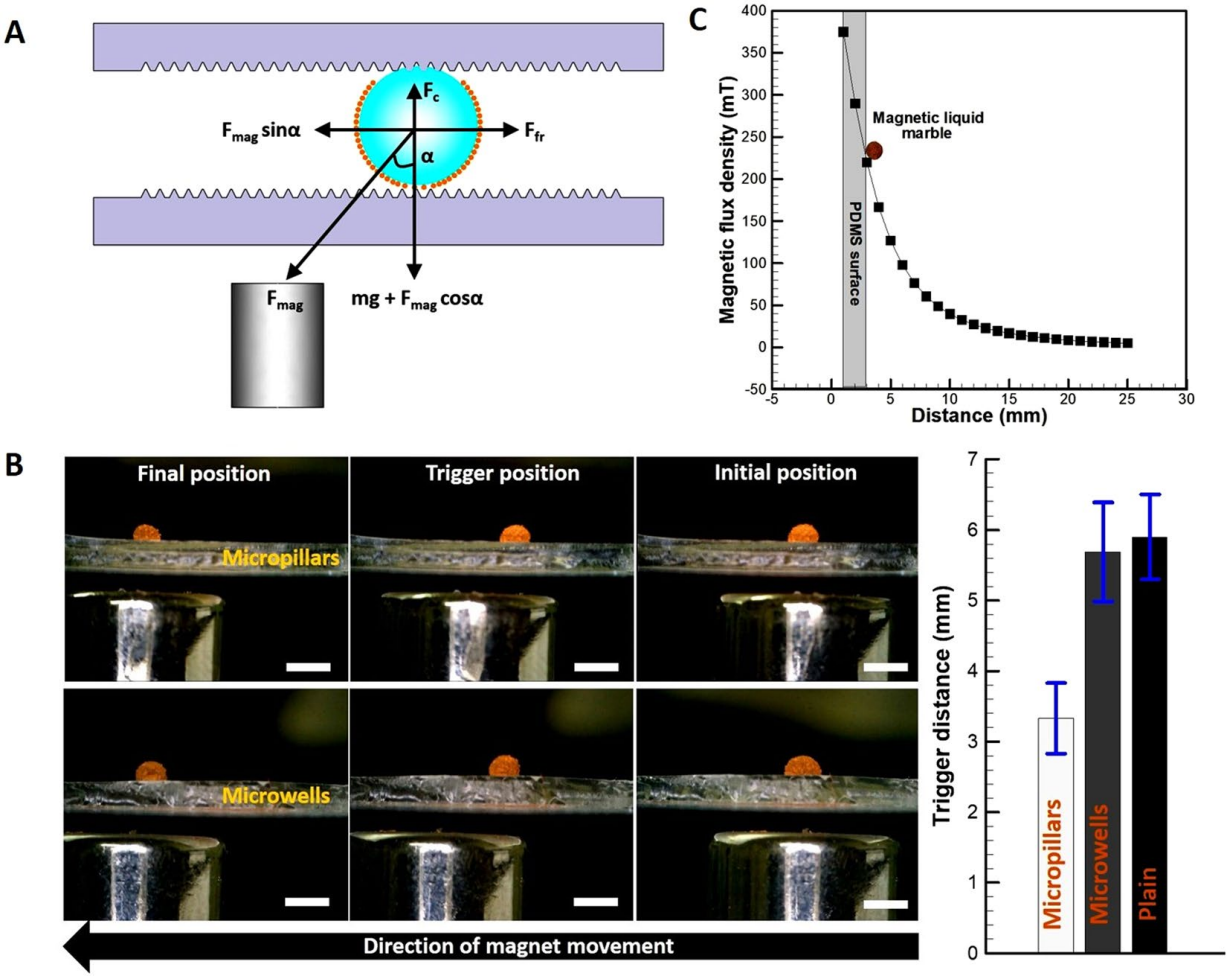
(**A)** Schematic of forces acting on a magnetic liquid marble (**B)** Experimental snapshots showing position of the magnet with respect to the magnetic liquid marble on micropillar- and microwell-surfaces. A variation in trigger distance on plain and textured PDMS surfaces is also shown (scale bar – 4 mm). (**C)** The magnetic flux density as a function of distance away from the NdFeB cylindrical magnet surface. Exact location of the magnetic liquid marble resting on the PDMS surface is also shown.

The balance of forces acting on a magnetic liquid marble inside the microdevice is schematically shown in Fig. 7A. As shown in Fig. 7A, the total frictional force (*F*_*fr* = _*F*_*Tfr*_ + *F*_*Bfr*_) constituting frictional force at the top (*F*_*Tfr*_) and bottom plates (*F*_*Bfr*_) acts on the magnetic liquid marble. When the magnet is placed right under the magnetic liquid marble, no horizontal force acts on the magnetic liquid marble. Since there is no component of force is present to overcome the total frictional force (*F*_*fr*_), the magnetic liquid marble remains stationary. However, with the movement of the magnet away from the magnetic liquid marble, a horizontal component of the force due to magnetic field (*F*_*mag*_sin*α*) will be generated as shown in Fig. 7A. This force will trigger the movement of the magnetic liquid marble. Further, the normal force (N) is balanced by the weight of the magnetic liquid marble (*mg*, where *m* is mass of the droplet along with the Fe_3_O_4_ nanoparticles and *g* is the acceleration due to gravity) and vertical component of the force due to magnetic field (*F*_*mag*_cos*α*). The force due to magnetic field on a droplet covered with ‘*N*’ number of particles is expressed as^29^:

2 $${F}_{mag}=\frac{N{\rm{\Delta }}\chi {V}_{m}}{{\mu }_{o}}(\nabla B)B.$$

where, *Δχ* is the difference in the magnetic susceptibility between the Fe_3_O_4_ nanoparticles and the surrounding medium, *V*_*m*_ is the volume of magnetic nanoparticle, *µ*_*o*_ is the permeability of vacuum (4π × 10^−7^, H/m), *B* is the magnetic flux density (*T*) and *∇B* is the gradient of magnetic field (T/m). Since the surrounding medium is air in the experiments, the difference in the magnetic susceptibility *Δχ* reduces to the magnetic susceptibility of the Fe_3_O_4_ nanoparticles. In addition, the microwells on the top plate cause the magnetic liquid marble to experience the force due to capillary pressure (*F*_*c*_) as shown in Fig. 7A. As the functionality of the top plates is premeditated, the selection of suitable topography for the bottom plate depends on reducing the traction on magnetic liquid marble. Therefore, three topographies such as plain, microwell- and micropillar-surfaces have been investigated to estimate the traction on magnetic liquid marble.

In order to trigger the movement of the magnetic liquid marble on a surface, the critical angle (*α*) which is a function of position of the magnet needs to be determined (see Fig. 7A). The experiments were conducted by placing a magnetic liquid marble (~2 µL) on the plain and textured PDMS surfaces. Figure 7B shows snapshots of different positions of the magnet below the micropillar- and microwell-surfaces holding the magnetic liquid marble. When the magnet was below the magnetic liquid marble, no movement was noticed. However, once the magnet was moved towards left as shown in the Fig. 7B, a movement was triggered at a certain distance. The triggered distance was measured to be different for the three surfaces examined as shown in plot of the Fig. 7B. Due to the less apparent contact area between the magnetic liquid marble and the surface, the triggered distance was observed to be less on the micropillar-surface, followed by the microwell-surface and plain surface. This situation is identical to rolling of a solid sphere on an inclined textured surface, which was examined recently by Ryu *et al*.^30^. The rotational and translational velocities of spheres were observed to be more on micropillar-surfaces than the microwell-surfaces, as the former surface offer lesser apparent contact area.

The threshold force of magnetic field (*F*_*mag*_sin*α*) to trigger the movement of the magnetic liquid marble was estimated for the plain and textured PDMS surfaces. The magnetic flux density as a function of distance away from the magnet top surface is shown in Fig. 7C. For a closely packed arrangement of the Fe_3_O_4_ nanoparticles on the surface of a droplet, with a contact angle of 90° at the water-air interface, the number of particles cloaked on a 2 µL droplet was calculated to be ~1 × 10^12^. The magnetic susceptibility of the magnetic nanoparticles was 1.1, as calculated from the slope of line fitted to the magnetization curve in Fig. 2C. The produced magnetic flux density and magnetic field gradient when the magnetic liquid marble was 3 mm away from the magnet were 219.89 mT and 61.59 T/m respectively. The estimated critical angle (based on the distance between the magnetic liquid marble and the magnet) and threshold force for the three surfaces investigated are listed in Table 1.

**Table 1:** Estimated values of critical angle (see Fig. 7A) and threshold force for three surfaces.

Surface	Critical angle (*α*)	Threshold force (µN)
Plain	57.4°	~3.80
Microwell-surface	56.4°	~3.76
Micropillar-surface	41.5°	~2.99

The value of threshold force indicates that the micropillar-surface offer the least resistance to the movement of the magnetic liquid marbles as compared to the plain and microwell-surfaces. As mentioned in section 3.2, the blunt conical shape imparts higher apparent contact angle to the micropillars as compared to other shapes such as cylindrical (flat-top or hemispherical-top) and parallelepiped^18^. This leads to a low apparent contact area between the micropillar-surface and the magnetic liquid marble, which prevents loss of magnetic nanoparticles from the periphery of the magnetic liquid marble. This, in turn, assists in the smooth movement of the magnetic liquid marble between the top and bottom plates, reducing the processing time for isolating biological entities in the microwell array. Therefore, micropillar topography has been selected for the bottom plate in the closed magnetic DMF device.

## Functioning of Closed Magnetic Dmf Platform

The closed magnetic DMF device was constructed by assembling the top and bottom plates containing micropillars and microwells, respectively. Initially, the textured PDMS films were cut into rectangular sections of 24 mm × 60 mm. The thickness of these surfaces was measured to be about 2 mm. The thickness was found to be slightly non-uniform over the surface due to undulations of the base mould i.e., lotus leaf. A 3 mm hole was punched on PDMS film containing microwells to aid the passage of magnetic liquid marbles between the top and bottom plates. The film containing microwells was then pasted on a rectangular coverslip (24 mm × 60 mm × 0.17 mm), which served as the top plate. Similarly, the film containing micropillars was pasted onto another rectangular coverslip, which served as the bottom plate. Thereafter, the sandwiched structure was completed by bonding the top plate with the bottom plate, using a spacer (double sided tape of 2 mm thick) as illustrated in Fig. 1.

**Figure 8:**
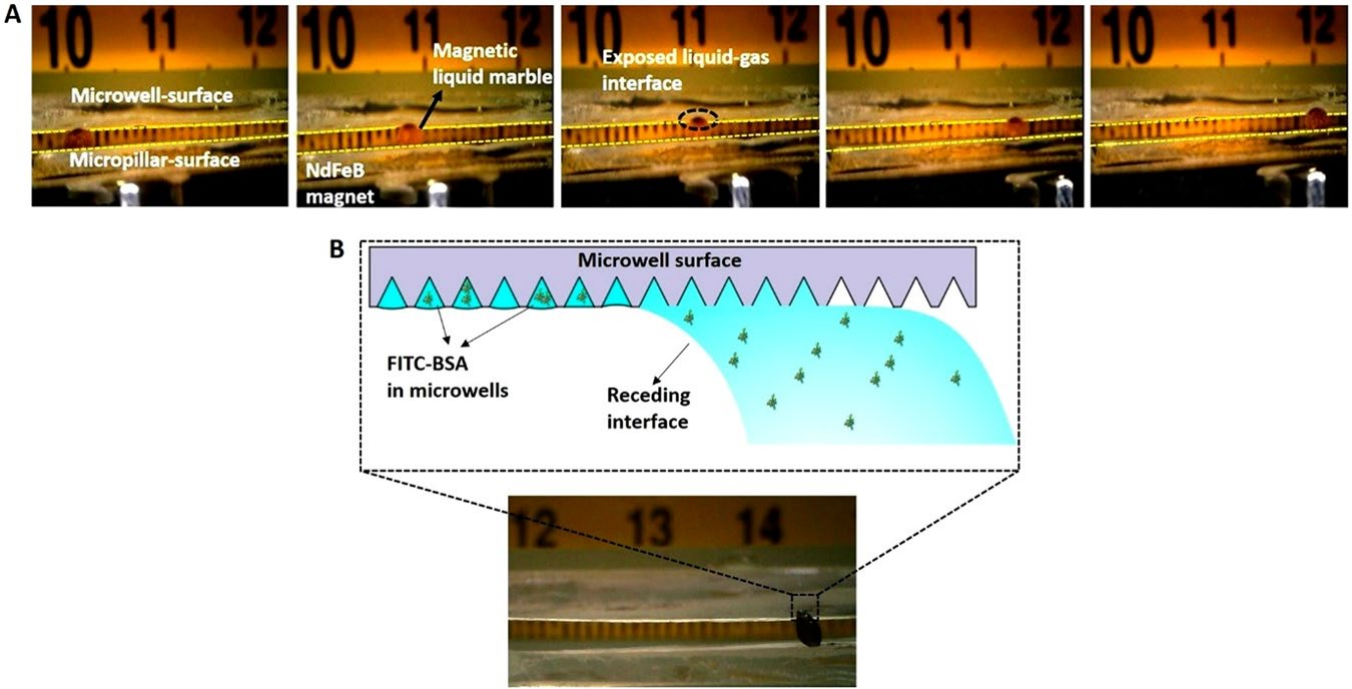
(**A)** Snapshots showing the movement of magnetic liquid marble in the sandwiched structure along with the shell opening to allow filling of protein solution in the microwells. (**B)** Entrapment of biological entities (FITC-BSA/microbeads/*E*. *coli*) in the microwells by the receding interface.

Once the construction of sandwiched structure was completed, a 2 µL DI water droplet containing biological entity of interest was pipetted onto a bed of Fe_3_O_4_ nanoparticles to form magnetic liquid marble. The magnetic liquid marble was then gently placed inside the chamber via the passage made on the top plate. Then, the cylindrical magnet was attached to a manual linear stage and positioned at an optimal distance (*D* = 1 mm) from the bottom plate for the near-frictionless movement and to allow the shell opening as well. The magnetic liquid marble was then allowed to displace by continuous back and forth motion of the magnet. Figure 8A depicts the experimental snapshots of magnetic liquid marble sandwiched between top and bottom plates. Both the shell opening and movement of the magnetic liquid marble is shown in the Fig. 8A. The biological entity inside the magnetic liquid marble was isolated in the microwells as the exposed area of the water-air interface recedes the top plate. Due to the varied wettability of upper (hydrophilic) and lower films (hydrophobic), a stick-slip motion of droplet was observed in the sandwiched structure. The exposed water-air interface leaves its footprint on the top plate containing microwell array by the receding meniscus (or interface) as shown in Fig. 8B. Further, the receding meniscus swept away excess biological sample from the area between microwells, thereby allowing the biological entities to be captured only in microwells. Once the meniscus leaves the microwell array, capillary forces hold the captured biological entities in place. Moreover, conical shape of the microwells help in uniform distribution of cells and protects the cells against the shear force acting during the loading in microwells^31,32^.

## Application of the Microdevice

In this section, the application of the developed closed magnetic DMF platform in isolation of biological entities in microwells will be discussed. Three different types of entities viz, microbeads, FITC-BSA and *E*. *coli* were isolated in microwells. In all the experiments, the sample of interest were encapsulated in magnetic liquid marbles of a volume of 2 µl and shuttled (number of back and forth movements of magnetic liquid marble for a distance of 10 mm) in the chamber.

### Isolation of microbeads in microwells

**Figure 9:**
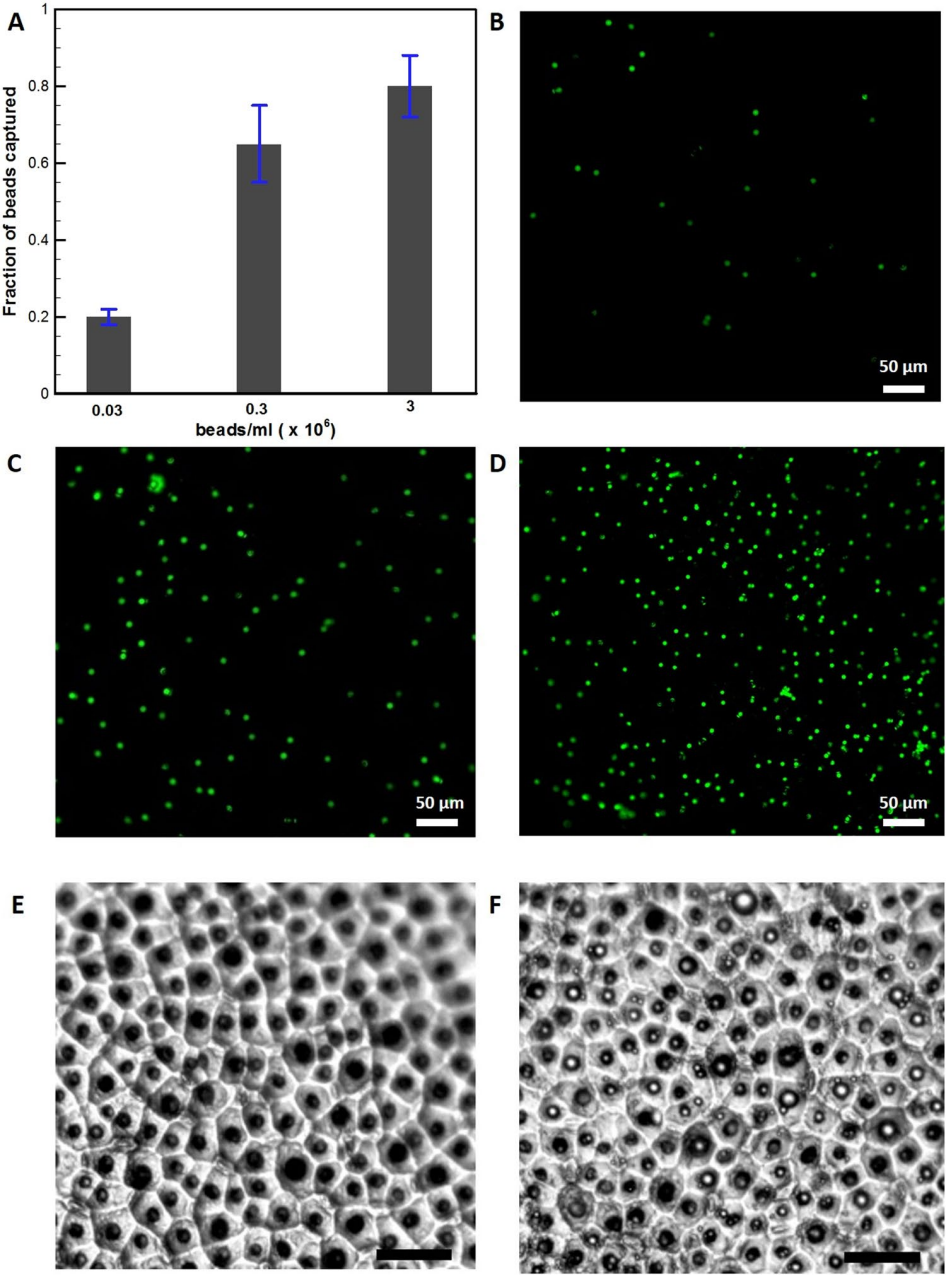
**(A**) The loading efficiency of microwells with respect to concentration of microbeads in magnetic liquid marble. Fluorescent micrographs showing digital quantification of microbeads isolated in microwells for a concentration of (**B)** 0.03 × 10^6^ beads/ml (**C)** 0.3 × 10^6^ beads/ml (**D)** 3 × 10^6^ beads/ml in magnetic liquid marble. Phase contrast image showing (**E)** empty microwells (**F)** microbeads in microwells (scale bar- 25 µm).

The usage of antibody-coated microspheres or microbeads for isolating low abundant protein biomarkers is gaining importance in measurement of proteins due to their ease of handling^9,11,33^. In addition, bead-based immunoassays improve isolation efficiency and avoid the need of time-consuming washing steps. In earlier attempts, the microbeads were isolated in microwells by either gravity or magnetic field-assisted. Here, we have made an attempt to isolate FITC-marked microbeads (6 µm diameter, Sigma Aldrich) using the magnetic liquid marbles. In the present experiments, the concentration of microbeads in the magnetic liquid marbles was varied in three steps viz., 0.03 × 10^6^ beads/ml, 0.3 × 10^6^ beads/ml and 3 × 10^6^ beads/ml to study the isolation efficiency of the microdevice. As the magnetic liquid marble traverses one cycle, the receding interface at the top plate allows the microbeads to get captured in the microwells. An increase in either microbeads concentration or shuttling cycles of the magnetic liquid marble will allow a large number of microbeads to be loaded into the microwells. Images were acquired immediately after performing each experiment with a 20× objective. The fraction of wells loaded with microbeads was calculated by dividing the number of events of the fluorescent signal from the microwells counted by the ImageJ software (US National Institutes of Health, http://rsb.info.nih.gov/ij/) by the total number of microwells. Three experimental replicates were performed for each concentration of microbeads. Figure 9A shows the fraction of single beads isolated in microwells at three different concentrations for 5 shuttling cycles. As it can be seen from the Fig. 9A, almost 80% of microwells were occupied by microbeads with an increase in concentration. Figure 9B,C and D show fluorescent images of isolated microbeads at three different concentrations. Similarly, Fig. 9E,F show bright field images of vacant and filled microwells (concentration of 3 × 10^6^ beads/ml) before and after isolation, respectively.

### Isolation of FITC-BSA and *E*. *coli* in microwells

**Figure 10:**
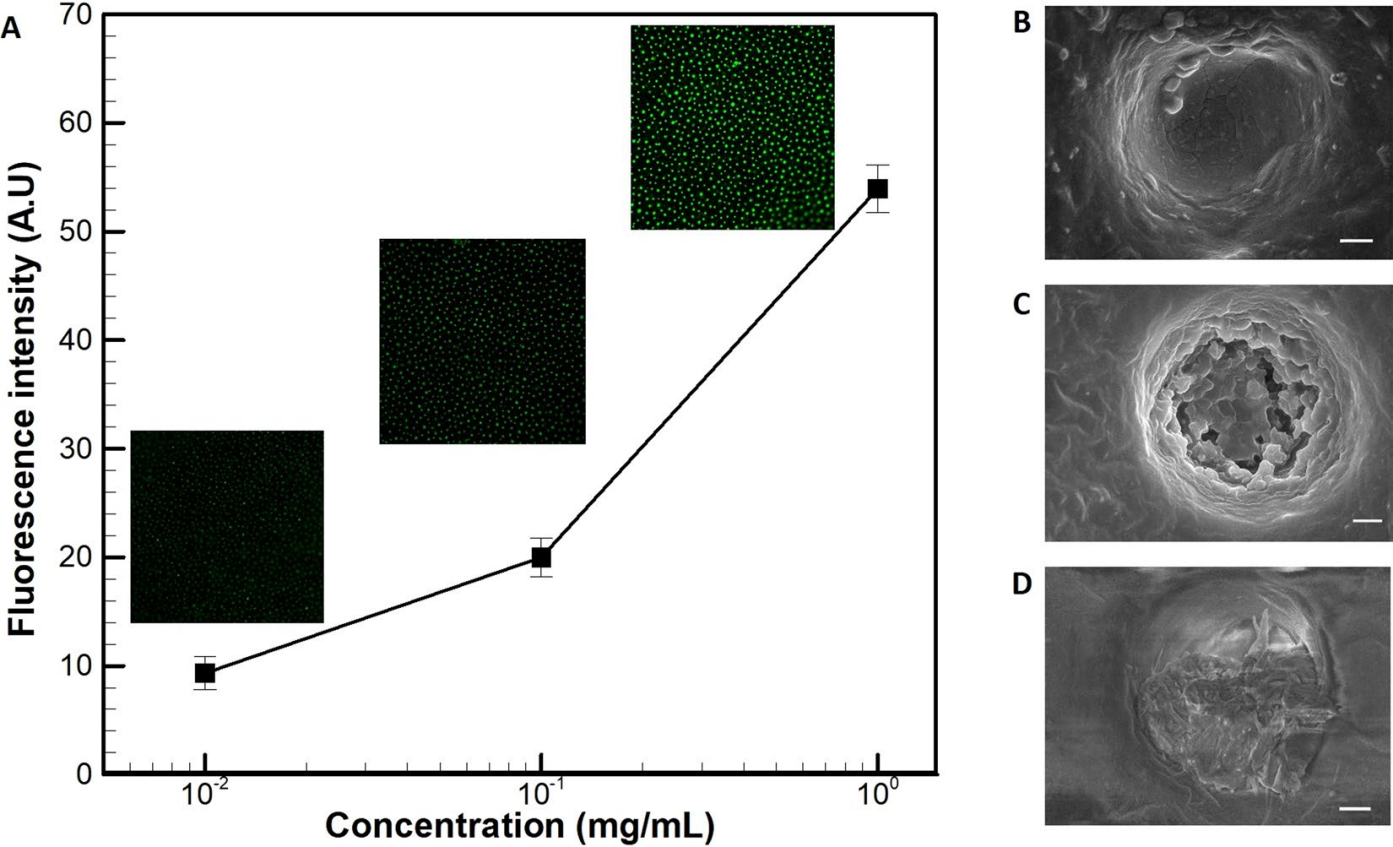
**(A)** Fluorescence signal measured with respect to the concentration of FITC-BSA in solution. FEG-SEM micrographs for (**B)** empty microwell (**C)** partially filled microwell at 0.1 mg/ml (**D)** completely filled microwell at 1 mg/ml (scale bar −2 µm).

In the subsequent experiments, isolation of low volume FITC-BSA was investigated by recording the fluorescent signal from the microwells. Magnetic liquid marbles with different concentrations of the FITC-BSA viz., 0.01, 0.1 and 1 mg/ml were prepared. The experiments were conducted in a similar manner as explained previously. The measurements were carried out for three experiment replicates. Around 20 microwells were randomly chosen from each image and fluorescent intensity was calculated by the ImageJ software. Figure 10A shows the variation of fluorescent intensity from the microwells with the concentration of FITC-BSA in magnetic liquid marbles. It is evident from the figure that an increase in concentration of the FITC-BSA resulted in improved fluorescent signal from the microwells. The corresponding fluorescent images are shown in the inset of Fig. 10A. The isolated FITC-BSA in the microwells was visualized through FEG-SEM. Figure 10B shows an image of unfilled microwell without any protein before the experiment. Figure 10C,D show the isolated FITC-BSA at 0.1 mg/ml and 1 mg/ml respectively, indicating that more amount of protein occupied the microwell with an increase in the concentration.

**Figure 11:**
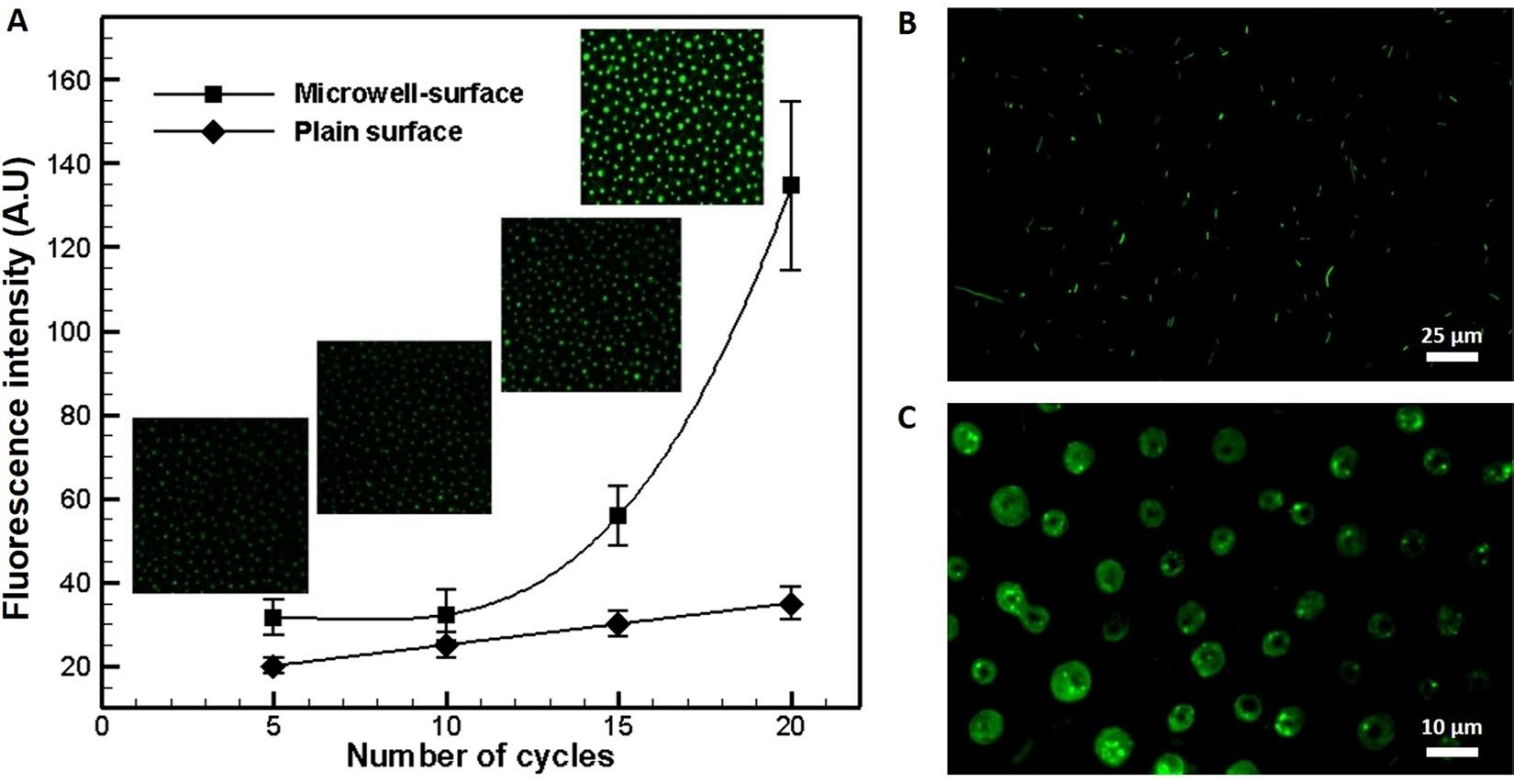
**(A)** Variation of fluorescent intensity with shuttling cycles of the magnetic liquid marble with a concentration of 1 mg/ml. Fluorescent intensity obtained on plain surface is also shown for reference. Fluorescent microscopy images of live *E*. *coli* bacteria (**B)** on pristine PDMS surfaces and (**C)** isolated in microwells.

Further, the effect of shuttling cycles of the magnetic liquid marble on filling of the microwells was analyzed. Figure 11A shows the variation of the fluorescent intensity with the shuttling cycles at four different levels: 5, 10, 15 and 20 for a concentration of 1 mg/ml of FITC-BSA in magnetic liquid marble. An exponential-like increase in fluorescent intensity with respect to shuttling cycles was observed. This can be attributed to the accumulation of FITC-BSA in the microwells with time, as the water-air interface exposed to the top plate more time with an increase in number of shuttling cycles. In a similar way, an overnight grown and fluorescently stained *E*. *coli* BL21 (DE3) culture was diluted and isolated as shown on a plain PDMS film (Fig. 11B). Then, the *E*. *coli* bacteria was encapsulated in the magnetic liquid marbles and isolated in microwells as shown in Fig. 11C. Here, the proposed detection method is based on fluorescent tagging of target biological entity for visualization. However, the non-fluorescent biological entities can also be detected by visualizing a secondary binding of biomolecules to a fluorescent dye^34^.

## Conclusions

The detection of low-abundant protein biomarkers is crucial for early-stage disease diagnostics, and therefore essential for clinical and biological applications. In addition, the current assays based on the average response of a population of cells do not reflect the individual cell characteristics. Therefore, isolation of such biological entities is an important step to study or quantify certain immunoresponses. In this work, we propose, fabricate and characterize a microdevice for isolating a multitude of biological entities in a microwell array. We have leveraged the advances in magnetic digital microfluidics, polymer replication, biomimetics and fluidics integration to develop the microdevice, which is of low-cost, power-free and simple in operation.

The microdevice exploits surface wettability and meniscus-induced patterning to isolate biological entities in the microwell array. The ease of individual component fabrication and reduced number of steps involved in its assembly makes the proposed microdevice free of costly equipments and technological skills. Further, the use of lotus leaf derived micropattern provides added cost benefit and additional functionalities. The system design also offers sufficient flexibility to realize application-specific microdevices by tailoring the micropatterns. The microdevice can either be manually operated or controlled using automation techniques. The proposed method presently has capability to capture biological entities of pure sample. However, it is also feasible to capture biological entities in impure samples by the same method, using molecule/protein specific antibodies tagged with differential flurochromes.

There are, however, some challenges associated with the current approach using magnetic liquid marbles, which are amenable. First, the magnetic liquid marble deforms with an increase in droplet volume, causing difficulty in movement inside the chamber. Thus, a correlation for displacement of the magnetic liquid marble as a function of shape, particle loading and magnetic forces would help customize devices for specific applications. Second, gravity acts against patterning of the biological entities encapsulated in magnetic liquid marbles in the current configuration. Yet, a loading efficiency of about 80% was achieved with the microbeads. An inverted design would further increase the loading efficiency of the microdevice. Further, the issues related to contamination of the biological sample during the formation and actuation of the magnetic liquid marbles need to be resolved for successful implementation of the microdevice.

The proposed closed magnetic microfluidic device should provide a useful way-forward towards development of high-throughput analysis techniques, with applications to drug-protein interaction, protein measurement, single-cell studies, among others, in a cost-effective manner.

## Materials and Methods

### Fabrication of textured surfaces

A garden-fresh lotus leaf was used as a mould to fabricate surfaces with microwells and micropillars. Essentially, the topography of lotus leaf consists of microbumps of the size of ~10 µm in height and ~5 µm in diameter, which are in turn is covered with hydrophobic wax. These textures along with low surface energy wax layer promote a high contact angle and low contact angle hysteresis, making them water-repellent. To replicate these textures over polymeric films, a two-step soft lithography procedure was adopted. Initially, the leaf was cut into 6 cm × 6 cm sections and bonded on to glass slides. Then, a polydimethylsiloxane (PDMS) base and curing agent (Sylgard 184, Dow Corning, USA) were mixed in a volume ratio of 10:1 and degassed. The mixture was then poured into the moulds and baked for over 24 hours to allow self-curing without aid of heating. The cured PDMS films were then peeled off from the moulds. In this step, a negative replica consisting of microwells was formed on the PDMS films. This negative replica served as a mould for replication of micropillars on the PDMS films. However, it is necessary to ensure that no bonding takes place between these PDMS films. To this end, the PDMS films containing microwells were treated with Trichloro(1H,1H,2H,2H-perfluorooctyl)silane (Sigma Aldrich) in a vacuum desiccator for 60 minutes. The mixture of PDMS base and curing agent in the same volume ratio was then poured into the silane-coated microwell-mould and it was allowed to cure for 24 hours, before peeling off.

### Surface modification

In the present work, oxygen plasma treatment was employed to modify the textured PDMS surface as it can render hydrophilicity to PDMS surfaces over relatively longer periods than the previously mentioned methods. Oxygen plasma treatment introduces polar functional groups such as silanol group (SiOH) on PDMS surfaces thereby changing its surface from hydrophobic to hydrophilic. In the present experiments, plasma power was kept constant at 70 W with an oxygen flow rate of 25 sccm, while optimizing the treatment times. The higher the treatment time, longer the retention time of hydrophilicity as reported previously^35^. It was observed that the optimal treatment time was 120 s, for a hydrophilicity retention time of 3 hours, which was well within the duration of the experiment.

### Synthesis of Fe_3_O_4_ nanoparticles

Fe_3_O_4_ nanoparticles were prepared by co-precipitation method. In this procedure, 10 ml of 0.5 M ferrous sulphate (FeSO_4_.7H_2_O, Merck Darmstadt, Germany) and 20 ml of 0.5 M ferric chloride (FeCl_3_.6H_2_O, Merck Darmstadt, Germany) were mixed in a glass beaker. To this solution, 60 ml of 12 M NH_4_OH (S.D. Fine Chemicals, Mumbai, India) was added drop wise with continuous stirring for 24 hours under nitrogen environment until the formation of a black precipitate. The precipitate was washed with 20 ml of double distilled water three times, to remove excess NH_3_ (S.D. Fine Chemicals, Mumbai, India).

### Instrumentation

The geometry and dimensions of the microwells and micropillars have been critically analyzed by an optical profilometer (Zeta-20, Zeta Instruments, USA) and an environmental scanning electron microscope (ESEM, Philips FEI Quanta-200, The Netherlands). The size and shape of the as-synthesized Fe_3_O_4_ nanoparticles was analyzed by transmission electron microscope (TEM, Philips CM200, The Netherlands). The phase purity of as-synthesized Fe_3_O_4_ nanoparticles was established by X-ray diffraction (XRD) patterns (PANalytical, PW3040/60, Netherlands) using Cu Kα (1.54 Å) radiation. The magnetic properties of the Fe_3_O_4_ nanoparticles were measured using a physical property measurement system (PPMS, Quantum Design, USA). The protein measurements were conducted with a Fluorescence microscopy (Leica microsystems, USA), UV spectroscopy (JASCO, V650, Japan) and imaging was carried out by field emission gun-scanning electron microscope (FEG-SEM, JEOL-7600F, Japan). The filling of microwells was imaged by confocal laser scanning microscope (Axio-Observer Z1 microscope, Zeiss, Germany). To characterize the surface wettability, equilibrium water contact angle was measured (GBX Digidrop, France) on the plain, micropillar-, and microwell- PDMS surfaces with 5 µL de-ionized (DI) water droplets. The images of movement of magnetic liquid marbles were acquired using a mobile imaging system (Dino-Lite digital microscope, 5MP, Taiwan). The magnetic flux density of an NdFeB cylindrical magnet (10 mm in diameter and 10 mm in length, Techtone magnetics, India) used in this study was measured by an in-house developed gauss meter.

### Labelling BSA with FITC

For protein isolation experiments, bovine serum albumin (BSA) was labelled with fluorescein isothiocyanate (FITC). A stock of 5 mg/ml BSA (Sigma Aldrich) was prepared in 0.1 M of sodium bicarbonate buffer, pH 9. In a separate step, FITC (Sigma Aldrich) was dissolved in Dimethyl sulfoxide (DMSO, Thermofisher) at 1 mg/ml. Then, for each 1 mL of protein solution, 50 μL of FITC solution was added very slowly in 5 mL aliquots while gently stirring the solution. After the required amount of FITC solution was added, the reaction was incubated in the dark for 8 hours at 4° C. Then, a 50 mM NH_4_Cl (Merck) was added and incubated for 2 hours at 4° C. The unbound FITC was separated from the conjugate by PD-10 desalting column.

### Bacterial culture

*E*. *coli* BL21 (DE3) colony, grown on Luria Agar plate, was inoculated in Luria Broth, Himedia (Himedia, Mumbai, India). The culture was incubated with shaking conditions (~150 rpm) at 37 °C for 12 hours. A 3 ml of bacterial culture was pelleted down by centrifuging the culture at 3000 rpm for 3 minutes. The pellet obtained was washed with 200 μl Phosphate buffered saline, pH 7.4. The washed pellet was resuspended in 500 μl Phosphate buffered saline. The LIVE/DEAD™ BacLight™ Bacterial Viability Kit (Thermofisher Scientific, USA) was used for staining of the bacteria as per manufacturer’s instructions. Briefly, 1.5 μl SYTO 9 dye and 1.5 μl Propidium iodide were mixed and added to the resuspended bacterial pellet and incubated in dark for 15 minutes. The culture was suitably diluted before isolation of the bacteria.

### Data acquisition

All the experiments were conducted in darkroom to prevent introduction of errors in the data. Initially, a negative control (only the device surface with buffer/DI water) was used to set the parameters of the microscope to ensure no background signal is captured. The fluorescent intensity of the samples was recorded after applying the same parameter values of exposure time, gain and intensity as that of the control.

## Electronic supplementary material

Supplementary information

## Data Availability

Supplementary information is provided with this manuscript.
