# Supplementary material for: A magnet-actuated biomimetic device for isolating biological entities in microwells
Source: Sci Rep. 2018 Aug 24;8:12717. doi: 10.1038/s41598-018-31274-z (PMC6109070; doi:10.1038/s41598-018-31274-z)
Supplement: Supplementary file 1 — Supplementary information [file 41598_2018_31274_MOESM1_ESM.pdf]

Supporting Information

A magnet-actuated biomimetic device for isolating biological entities in microwells

Himani Sharma[1], Kimberley John[2], Anvesh Gaddam[1], Ambuja Navalkar[2], Samir K. Maji[2], Amit Agrawal[1]

[1]Department of Mechanical Engineering, Indian Institute of Technology Bombay, Powai, Mumbai 400076, India.

[2]Department of Biosciences and Bioengineering, Indian Institute of Technology Bombay, Powai, Mumbai 400076, India.

Experiments to validate the mechanism of imbibition in microwells

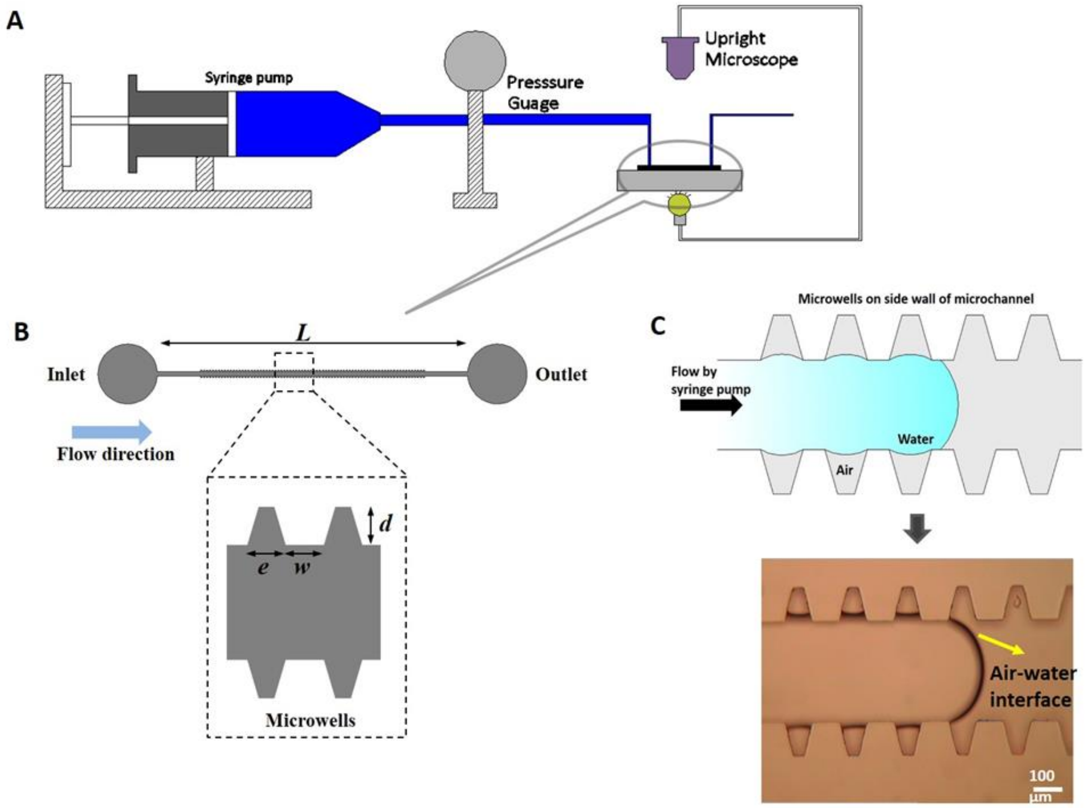

Figure S1: Imbibition mechanism in microwells of different wettability (A) A schematic of the experimental set-up. (B) The microchannel containing microwells with the nomenclature. The microwells in the microchannels were fabricated in such a way to maintain the shape of lotus

leaf-based microwells. To facilitate proper visualization of dynamics of the air-water interface, the microwells were fabricated with larger dimensions (e = w = d =100 µm). (C) Images showing flow of water inside the microchannel.

Time-dependent imbibition process in micowells

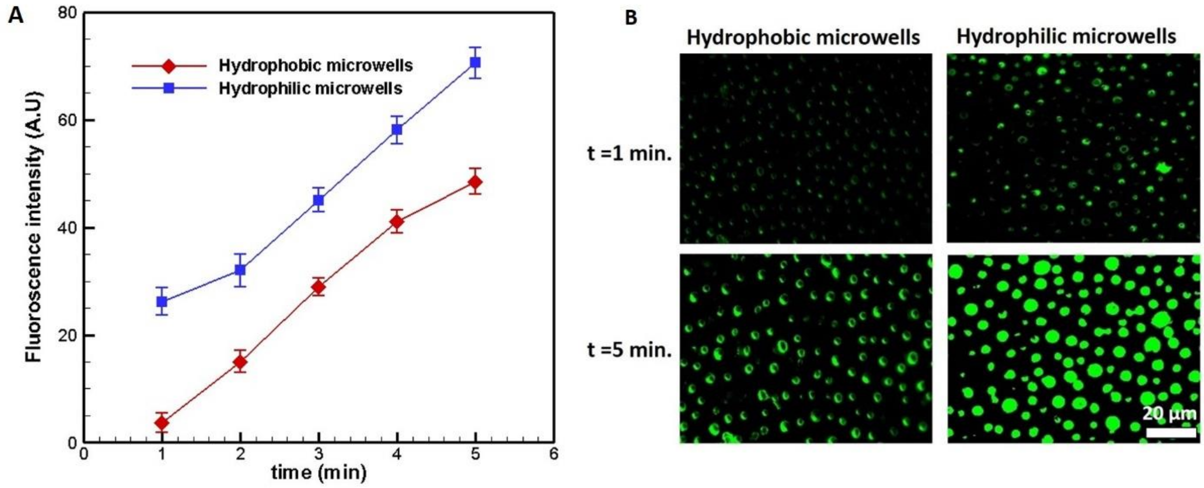

Figure S2: (A) Time-dependent variation of fluorescent intensity in microwells of different wettability indicating imbibition process. (B) The corresponding fluorescent micrographs at different time intervals.

Surface coverage of droplet with nanoparticles

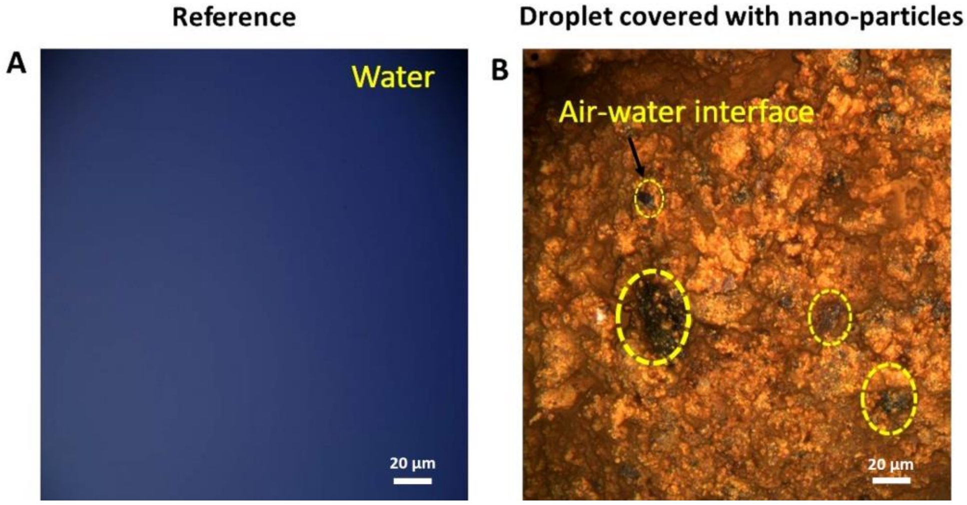

Figure S3: Micrographs of surface of the magnetic liquid marble acquired using an optical profilometer. (A) Before cloaking the droplet with magnetic nanoparticles (B) Covering of the droplet with magnetic nanoparticles.
